# pH-Sensitive Amphiphilic Diblock Polyphosphoesters with Lactate Units: Synthesis and Application as Drug Carriers

**DOI:** 10.3390/ijms25084518

**Published:** 2024-04-20

**Authors:** Kasumi Mochizuki, Violeta Mitova, Kimiko Makino, Hiroshi Terada, Issei Takeuchi, Kolio Troev

**Affiliations:** 1 Faculty of Pharmaceutical Sciences, Tokyo University of Science, 2641, Yamazaki, Noda 278-8510, Chiba, Japan; ksm.mochizuki@gmail.com (K.M.); makino@rs.noda.tus.ac.jp (K.M.); teradah@rs.noda.tus.ac.jp (H.T.); itakeuchi@jiu.ac.jp (I.T.); 2 Institute of Polymers, Bulgarian Academy of Sciences, 1113 Sofia, Bulgaria; mitova@polymer.bas.bg; 3 Faculty of Pharmaceutical Science, Josai International University, 1 Gumyo, Togane 283-8555, Chiba, Japan

**Keywords:** polyphosphoesters, amphiphilic polymers, micelles, drug delivery

## Abstract

pH-sensitive amphiphilic diblock polyphosphoesters containing lactic acid units were synthesized by multistep one-pot polycondensation reactions. They comprise acid-labile P(O)-O-C and C(O)-O-C bonds, the cleavage of which depends on the pH of the medium. The structure of these copolymers was characterized by ^1^H, ^13^C {H}, ^31^P NMR, and size exclusion chromatography (SEC). The newly synthesized polymers self-assembled into the micellar structure in an aqueous solution. The effects of the molecular weight of the copolymer and the length of the hydrophobic chain on micelle formation and stabilityand micelle size were studied via dynamic light scattering (DLS). Drug loading and encapsulation efficiency tests using doxorubicin revealed that hydrophobic drugs can be delivered by copolymers. It was established that the molecular weight of the copolymer, length of the hydrophobic chain and content of lactate units affects the size of the micelles, drug loading, and efficiency of encapsulation. A copolymer with 10.7% lactate content has drug loading (3.2 ± 0.3) and efficiency of encapsulation (57.4 ± 3.2), compared to the same copolymer with 41.8% lactate content (1.63%) and (45.8%), respectively. It was demonstrated that the poly[alkylpoly(ethylene glycol) phosphate-*b*-alkylpoly(ethylene glycol)lactate phosphate] DOX system has a pH-sensitive response capability in the result in which DOX was selectively accumulated into the tumor, where pH is acidic. The results obtained indicate that amphiphilic diblock polyphosphoesters have potential as drug carriers.

## 1. Introduction

Polyphosphoesters, based on poly(ethylene glycol) (PEG), are especially attractive advanced reactive and functional polymers due to the following advantages [1]: (i) they are highly reactive; (ii) they are water-soluble; (iii) the drug-carrying capacity is determined by the highly reactive P-H group in each of the repeating units; (iv) the chemical immobilization of drugs proceeds under mild conditions; (v) the presence of highly polar P=O group in the repeating units affords the possibility for physical immobilization of drugs; (vi) possibility of hydrophilic/hydrophobic balance control; (vii) they can be regarded as biodegradable (enzymes are the dominant component in the degradation process) and biocompatible synthetic polymers; (viii) they can be designed to have nontoxic building blocks; (ix) they can be administrated over a wider molecular weight range because, after hydrolysis, the low molecular PEG and phosphoric acid will be safely excreted; (x) easy to prepare in an industrial scale; (xi) they are low toxicity (IC50 1000 mg/kg). 

Polycondensation as a process for the preparation of amphiphilic polyphosphoesters has significant advantages compared to the polymerization process, namely (i) different starting hydroxyl-containing compounds can be used; (ii) synthesis can proceed without a catalyst; (iii) there is no need for the purification of the reaction product; (iv) degraded products can be designed in advance; (v) copolymers can be obtained; and (vi) there are commercially available starting monomers. Poly(alkylene H-phosphonate)s offers a unique opportunity to introduce various modifications at the phosphorus center through different reaction schemes [2,3,4,5,6,7,8,9,10,11]. Poly(oxyethylene H-phosphonate)s are multifunctional drug carriers, capable of converting into (i) hydrophilic; (ii) hydrophobic; (iii) amphiphilic, micelles, and (iv) stimuli-responsive poly(alkyloxyethylene phosphate)s. The drug can be carried (v) chemically (covalent bond), due to the presence of a highly reactive P-H group [12]; (vi) via ionic bonds (P-OH group) [12,13]; (vii) physically, due to the presence of a strong proton acceptor—P=O group [14], and via micelles [15,16]. It was discovered that polyphosphoesters had a stealth effect comparable to PEG [17,18]. By enhancing pharmacokinetics including blood circulation, biodistribution, and tissue targeting, the stealth effect is crucial in enabling nanomaterials for drug delivery applications. This frequent pharmacokinetics behavior of nanomaterials—dose-dependent nonlinear pharmacokinetics due to saturating or decreasing reticuloendothelial system (RES) bio-clearance—is referred to as the “pseudo-stealth effect” [19].

Micelles as a drug carrier have attracted significant attention in the targeted delivery of anticancer drugs. The polymeric micelle can efficiently accommodate the hydrophobic drug simply via physical entrapping (solubilization) in its hydrophobic core so that some advantages can be gained: (i) elimination of drug side effects; (ii) protection of drug molecules against possible degradation in particular media (pH, temperature); (iii) increasing the aqueous solubility of hydrophobic insoluble drugs; and (iv) control the drug release rate [20]. Nevertheless, unmodified micelles showed slow drug release preventing sufficient drug accumulation in cancer cells. Consequently, upgraded micelles with stimuli-sensitive (light, pH, temperature, ultrasound, and magnetic field) polymers were developed [21,22,23,24,25,26,27,28]. Different strategies have been used to release drugs from the carriers in the tumor’s acidic microenvironment [29]. For instance, pH labile chemical bonds such as hydrazone [30], Schiff base [31], acetal [32], ketal [33], ester [34], amide [35], amine [36], carboxyl [37], and ether [38] have been used for targeting tumor cells. Another mechanism of drug release involves the protonation of the hydrophobic core of the micelle below its pKa resulting in repulsion between polymer chains, destabilization, and eventually unloading of the drug in acidic cancer tissues [39]. 

pH-responsive DDSs have gained popularity since the pH in diseased tissues such as cancer, bacterial infection, and inflammation differs from a physiological pH of 7.4 and this difference could be harnessed for DDSs to release encapsulated drugs specifically to these diseased tissues [40]. The pH value in normal physiological conditions is 7.2–7.4, while the pH value in tumor tissues is about 6.5–6.8, and the pH value in tumor cells is about 5.0–5.5 [41]. Even the pH values of endosomes and lysosomes in cancer cells are low at 5.0–6.0 and 4.0–5.0, respectively [41]. Particularly, pH-responsive nanoparticles have emerged as an effective antitumor drug delivery system to release anticancer drugs selectively and rapidly in acidic tumoral tissue and cells [42]. In the present study, the chemical structure and composition of the diblock polyphosphoesters containing lactic acid units with acidic labile bonds in the main and the side chains, especially the effect of the molar ratio between the two blocks on micelle formation, drug loading, encapsulation efficiency, and drug release were investigated.

## 2. Results and Discussion

### 2.1. Microwave Synthesis of Poly(ethylene glycol)lactate

**Scheme 1:**
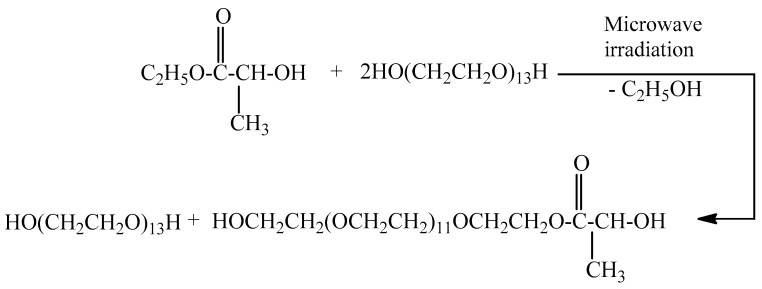
Synthesis of poly(ethylene glycol 600)lactate.

Poly(ethylene glycol)lactate was prepared via transesterification of ethyl lactate with poly(ethylene glycol 600) (Scheme 1). The reaction was carried out at a molar ratio of 1:2 at microwave conditions. The structure and composition of the reaction product were confirmed by ^1^H and ^13^C{H}NMR spectroscopy. In the ^1^H NMR spectrum (Figure S1) after 18 h of heating, there is no signal for C*H*_3_CH_2_O protons at 1.32 ppm. This revealed that the reaction product did not contain a free ethyl lactate. The two doublets at 1.43 and 1.428 ppm in the ^1^H NMR spectrum can be assigned to C*H*_3_CH-protons. Obviously, poly(ethylene glycol)lactate consists of the racemic mixture of levo and dextro forms. The multiplet in the area of 4.28 to 4.34 ppm can be assigned to CH_3_C*H*- and CH_2_C*H*_2_OC(O) protons. This signal for the CH_3_C*H*-proton has to be a quartet; those for CH_2_C*H*_2_OC(O) protons is a triplet but as the result of overlapping, the signal appears as a multiplet.

In the ^13^C{H}NMR spectrum (Figure S2), the signal at 14.21 ppm for the *C*H_3_CH_2_O carbon atom disappears. The signals at δ ppm are as follows: 20.34, 61.64, 64.40, 66.72, 68.83, 70.28, 72.48, and 175.51 can be assigned to the carbon atoms of methyl group *C*H_3_CH-, HO*C*H_2_-, CH_3_*C*H-, C(O)O*C*H_2_-; -C(O)OCH_2_*C*H_2_O-, HOCH_2_CH_2_O*C*H_2_-, -(O*C*H_2_*C*H_2_O)-, HOCH_2_*C*H_2_O-, *C*=O, respectively. Data from ^13^C{H} NMR spectroscopy confirm the structure of the reaction product. At a poly(ethylene glycol)lactate yield of 94.0%, the reaction mixture contains the following: poly (ethylene glycol)lactate 27.8 g, 0.041 mol, 49.3%; PEG 600 28.6 g, 0.048 mol, 50.7%. The use of a microwave reactor made it possible to avoid the use of a catalyst, eliminating the need for purification and successfully increasing the yield. The molar ratio between poly (ethylene glycol)lactate and PEG 600 is 1:1.2. The proposed method for the synthesis of poly(ethylene glycol)lactate allows varying the molar ratio between ethyl lactate and PEG to synthesize copolymers with different lactic acid content.

### 2.2. Synthesis of Poly[poly(ethylene glycol) H-phosphonate]-b-[poly(ethylene glycol)lactate H-phosphonate]

**Figure 1:**
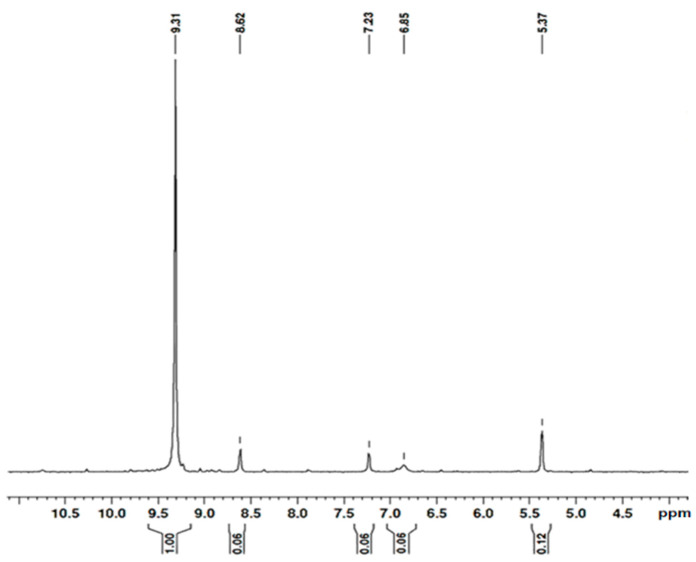
^31^P{H}-NMR spectra of poly[poly(ethylene glycol) H-phosphonate-*b*-poly(ethylene glycol)lactate H-phosphonate] in CDCl3.

**Scheme 2:**
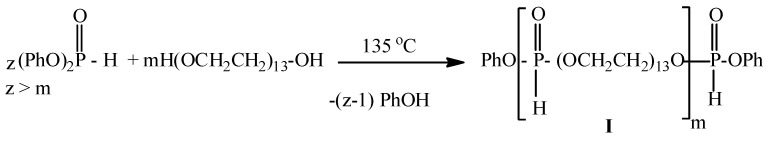
Interaction of diphenyl H-phosphonate with poly(ethylene glycol).

It is known that the oxygen atom of the secondary hydroxyl group is a weaker nucleophile compared to the oxygen atom of the primary hydroxyl group. On the other hand, it is known that diphenyl H-phosphonate is more reactive compared to dialkyl esters of H-phosphonic acid in transesterification reactions [1]. In this connection, we decided to use diphenyl H-phosphonate as a starting monomer for the preparation of poly[poly(ethylene glycol) H–phosphonate]-*b*-[poly(ethylene glycol)lactate H-phosphonate] using poly(ethylene glycol 600) and poly(ethylene glycol)lactate as a dihydroxy compound. To the reaction mixture (see Section 3.3) was added diphenyl H-phosphonate. The reaction was carried out at temperatures of 135 °C for 6 h, 160 °C for 4 h, and 185 °C for 3 h, vacuum 0.6 mm Hg. In the ^1^H NMR spectrum of the reaction product (see Figure S3), there are signals at 1.51 ppm, d, ^3^J(H,H) = 6.83 Hz, -POCH(C*H*_3_)C(O)-; 1.52 ppm, ^3^J(H,H) = 6.83 Hz, POCH(C*H*_3_)C(O)-(two diastereoisomers); 3.57–3.65, m, -OC*H*_2_C*H*_2_OP(O)(H)OCH_2_CH_2_-; 4.19–4.22 ppm, m, POC*H*(CH_3_)-protons; 6.75–6.79 and 7.10–7.24 ppm for aromatic protons; there are five types of P-H protons at δ = 6.89 ppm, integral intensity (II 1.0), d, ^1^J(P,H) = 720.0 Hz, which can be assigned to a P-H proton in OCH_2_OP(O)(*H*)OCH_2_ in the repeating units [15]; δ = 6.79 ppm, (II 0.06), d, ^1^J(P,H) = 732. 0 Hz for –P-H proton in CH_2_OP(O)(*H*)OCH(CH_3_)-; δ = 7.09 ppm, (II 0.06), d, ^1^J(P,H) = 732.0 Hz for –P-H proton in CH_2_OP(O)(*H*)OCH(CH_3_)-; δ = 7.10 ppm, (II 0.13) d, ^1^J(P,H) = 738.0 Hz for P-H proton in HOP(O)(*H*)OCH_2_-; δ = 7.41 ppm, (II 0.22), d, ^1^J(P,H) = 744.0 Hz for P-H proton in PhOP(O)(H)OCH_2_-. The ^31^P{H}NMR spectrum (Figure 1) of the reaction product shows five types of phosphorus atoms at δ = 9.31 ppm with integral intensity (II) = 1.00; 8.62 ppm, II = 0.06; 7.32 ppm, II = 0.06; 6.85 ppm, II = 0.06; 5.37 ppm, II = 0.12. The signal at δ = 9.31 ppm in the ^31^P NMR spectrum (Figure S4) is a doublet of quintets with ^1^J(P,H) = 737.6 Hz, and ^3^J(P,H) = 10.0 Hz. This signal can be assigned to the phosphorus atom with the following substituents -CH_2_O*P*(O)(H)OCH_2_-; the signal at 8.62 ppm appears as a doublet of quartets with ^1^J(P,H) = 751.98 Hz, and ^3^J(P,H) = 7.47. This signal can be assigned to the phosphorus atom with the following substituents -CH_2_O*P*(O)(H)OCH(CH_3_)C(O)-. The second doublet of quartets at 7.23 ppm with ^1^J(P,H) = 747.0 Hz, and ^3^J(P,H) = 7.47 can be assigned to the phosphorus atom with the same substituents as in the case of the first doublet of quartets. These data revealed that poly(ethylene glycol)lactate exists as a racemic mixture of D- and L-lactides [43]. It is a two-diastereoisomer. The ratio between the integral intensity of the signals at 9.31 ppm, 8.62 ppm, and 7.32 ppm is 1:0.12. The signals at 6.85 ppm which appear as a doublet of triplets with ^1^J(P,H) = 722.41 Hz can be assigned to the phosphorus atom in the end group HO*P*(O)(H)OCH_2_CH_2_; those at 5.37 ppm are a doublet of triplets with ^1^J(P,H) = 744.5 Hz, and ^3^J(P,H) = 9.96 Hz can be assigned to the phosphorus atom in the end group PhO*P*(O)(H)OCH_2_CH_2_. Based on the data from ^1^H and ^31^P NMR spectroscopy, the molar ratio between starting monomers, and the presence of a secondary hydroxyl group, we assume that the transesterification of with diphenyl H-phosphonate with poly(ethylene glycol) and poly(ethylene glycol)lactate proceeds according to the following Scheme 2.

**Scheme 3:**
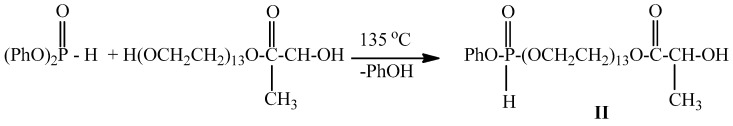
Interaction of diphenyl H-phosphonate with poly(ethylene glycol)lactate.

At this temperature, the primary hydroxyl groups will mainly participate in the transesterification reaction, i.e., diphenyl H-phosphonate will be in excess with respect to PEG. Oligo[ poly(ethylene glycol) H-phosphonate]s **I** with end phosphonates groups will be obtained. Transesterification of diphenyl H-phosphonate with poly(ethyl glycol)lactate results in the formation of phenylpoly(ethylene glycol)lactate H-phosphonate **II** (Scheme 3).

**Scheme 4:**
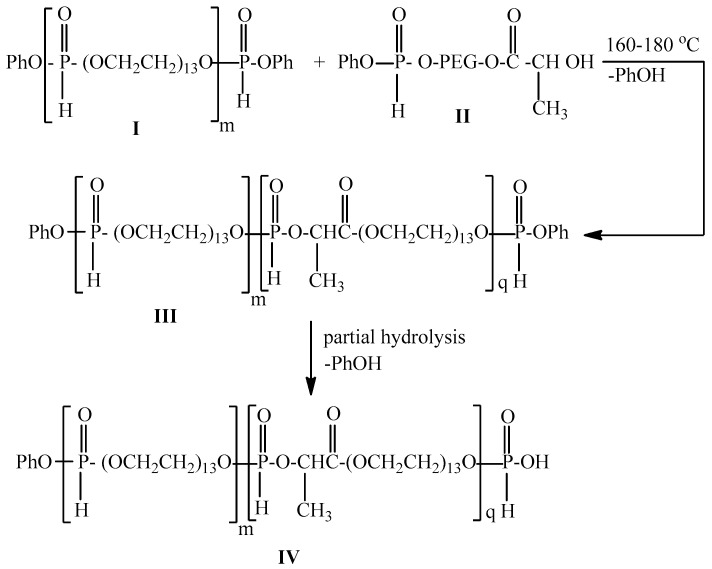
Synthesis of poly[poly(ethylene glycol) H-phosphonate-*b*-poly(ethylene glycol)lactate H-phosphonate].

In the temperature range of 160–185 °C, the secondary hydroxyl group of **II** will also participate in a transesterification reaction. The reaction between **I** and **II** will lead to the formation of polymer **III** (Scheme 4). The ratio between the integral intensities of the phosphorus in the end group and the phosphorus atom in the repeating unit is 1:0.06, i.e., *m* is equal to 16 (Figure 1). The ratio between the phenoxy group and the lactate-containing units is 0.12:0.06, i.e., *q* is 2. Molecular weight (Mw) based on ^31^P{H} NMR spectrum is (*m* = 16, Mw of repeating unit 646 g/mol; *q* = 2, Mw of repeating unit 718 g/mol) 11,772 g/mol. GPC gives 11,179 g/mol, PDS = 1.33. The hydrophilicity of the poly[poly(ethylene glycol) H-phosphonate-*b*-poly(ethylene glycol)lactate–H phosphonate] **IV** is determined by the poly(ethylene glycol), the phosphoryl group, which is a strong proton acceptor [44], and by the carbonyl group of lactic acid, which is a proton acceptor, too. By varying the ratio between *m* and *q*, we can control the hydrophilicity of the copolymer. 

### 2.3. One-Pot Synthesis of Poly[alkylpoly(ethylene glycol) phosphate-b-alkylpoly(ethyleneglycol)lactate phosphate]s

#### One-Pot Synthesis of Poly[hexadecylpoly(ethylene glycol) phosphate)-*b*-hexadecyl-poly(ethylene glycol)lactate phosphate]

**Scheme 5:**
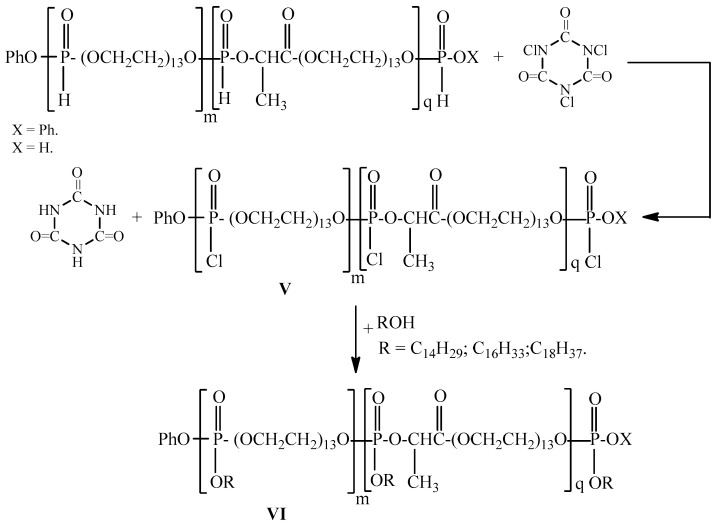
Synthesis of poly[alkylpoly(ethylene glycol) phosphate-*b*-alkylpoly(ethylene glycol)lactate phosphate]s.

Poly[(alkylpoly(ethylene glycol) phosphate-*b*-alkylpoly(ethylene glycol)lactate phosphate] was obtained via one-pot synthesis avoiding a lengthy separation process and purification of the intermediate chemical compound. For this purpose, poly[poly(ethylene glycol H-phosphonate-*b*-poly(ethylene glycol)lactate H-phosphonate] was converted into the corresponding poly[poly(ethylene glycol) chlorophosphate-*b*-poly(ethylene glycol)lactate chlorophosphate] via treatment with trichloroisocyanuric acid (Scheme 5). ^31^P{H}NMR spectrum of the reaction product (Figure S5) after 14 h heating revealed that the signals for the phosphorus atoms of poly[poly[(ethylene glycol) H-phosphonate-*b*-poly(ethylene glycol)lactate H-phosphonate] disappear and five new signals at δ (ppm) appear: 5.46; 4.95; 4.36; 0.24 and −0.08. These signals can be assigned to the phosphorus atoms of the poly[poly[poly(ethylene glycol) chlorophosphate-*b*-poly(ethylene glycol)lactate chlorophosphate]s (**VI**). The signal at δ = 5.46 ppm can be assigned to the phosphorus atom in the repeating units with the following substituents -CH_2_OP(O)(Cl)OCH_2_- [11]. Signals at δ = 4.95 and δ = 4.36 can be assigned to phosphorus atoms with the following substituents -CH_2_O-*P*(O)(Cl)OCH(CH_3_)-. The chirality of the CH carbon atom is a reason for the appearance of two signals. Signals at δ = 0.24 and −0.08 ppm can be assigned to phosphorus atoms with the following substituents: HO*P*(O)(Cl)OCH_2_- and PhO*P*(O)(Cl)OCH_2_, respectively.

After completion of the oxidation reaction, a solution of 1-hexadecanol in diethyl ether was added to the reaction product. The formation of poly[hexadecylpoly(ethylene glycol) phosphate-*b*-hexadecylpoly(ethylene glycol)lactate phosphate] was controlled by ^31^P{H} NMR spectroscopy. The reaction is stopped when signals for phosphorus atoms of poly[poly(ethylene glycol) chlorophosphate-*b*-poly(ethylene glycol)lactate chlorophosphate] disappear (Figure S6) and new signals appear at δ (ppm) −0.75; −1.63; −5.94, and −12.54, which are characteristic for phosphate structures -CH_2_O-*P*(O)(OR)OCH_2_- and -CH_2_O-*P*(O)(OR)-OCH(CH_3_)- and pyrophosphate structures (−12.54 ppm) [45]. In the ^1^H NMR spectrum (Figure S7), there is no signal at 11 ppm, characteristic of the P-OH proton, which revealed that the degree of alkylation reaction is almost quantitative. Using a one-pot synthesis strategy, we synthesized poly[tetradecylpoly(ethylene glycol) phosphate-*b*-tetradecylpoly(ethylene glycol)lactate phosphate] and poly[octadecylpoly(ethylene glycol) phosphate-*b*-octadecylpoly(ethylene glycol)lactate phosphate]. The application of a one-pot synthesis strategy for the preparation of poly[alkylpoly(ethylene glycol) phosphate-*b*-alkylpoly(ethylene glycol)lactate phosphate]s has a significant advantage because chlorophosphate group P-Cl is highly reactive and extremely sensitive to moisture, so purification requires an absolutely dry atmosphere and dry chemicals. Using a one-pot synthesis strategy, poly[alkylpoly(ethylene glycol) phosphate-*b-*alkylpoly(ethylene glycol)lactate phosphate]s were obtained in situ, without isolation of the corresponding poly[poly(ethylene glycol) chlorophosphate-*b*-poly(ethylene glycol)lactate chlorophosphate]s. 

### 2.4. Self-Assembly of Poly[alkylpoly(ethylene glycol) phosphate-b-alkylpoly(ethylene glycol)lactate phosphate]s and Particle Size Distribution and Drug Loading and Encapsulation Efficiency for Doxorubicin

Doxorubicin (DOX) is a type of anticancer drug useful as a drug that exerts various effects on conditions such as malignant lymphoma, breast cancer, and gastric cancer. However, it also has the disadvantage of being highly cytotoxic and causing many side effects such as myocardial failure, heart failure, and anaphylactic shock, so reducing these side effects is an issue. In this paper*,* the polymer type was symbolized as follows: poly[tetradecylpoly(ethylene glycol phosphate)-*b*-poly[tetradecylpoly(ethylene glycol)lactate phosphate]-**C14**; poly[hexadecylpoly(ethylene glycol) phosphate)-*b*-poly[hexadecylpoly(ethylene glycol)lactate phosphate]-**C16**; poly[octadecylpoly(ethylene glycol) phosphate)-*b*-poly[octadecylpoly(ethylene glycol)lactate phosphate] **C18**. 

#### 2.4.1. Particle Size Distribution

**Figure 2:**
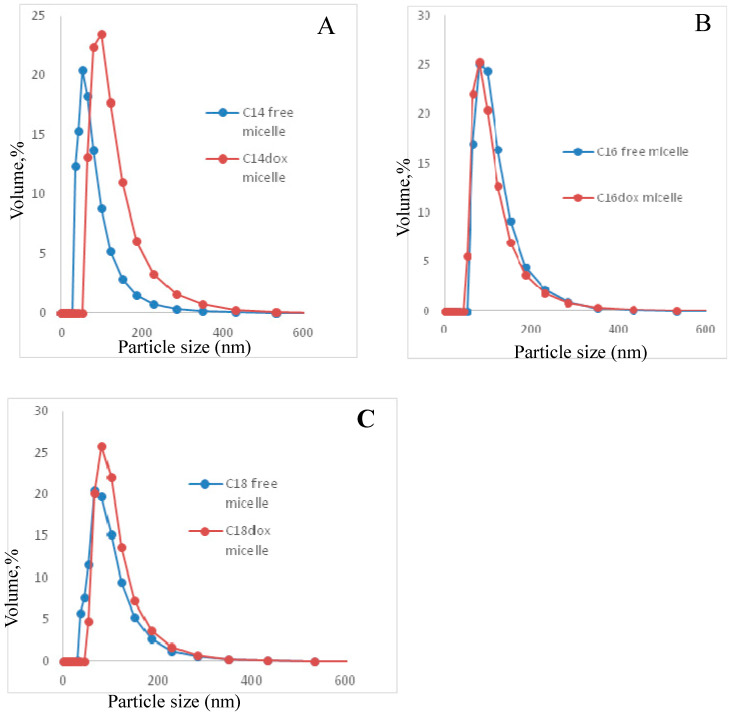
Particle size distribution of C14 (**A**), C16 (**B**), C18 (**C**) (*n* = 3).

Figure 2 and Table 1 show the particle size distribution of empty micelles and DOX-encapsulating micelles in PBS solution. 

The results indicate that the newly synthesized polymers self-assembled into the micellar structure in an aqueous solution and micelles show monodisperse peaks. Micelle size increases with the increasing molecular weight of the polymer and hydrophobicity. It was established that an increase in molecular weight and hydrophobic components of the diblock copolymer produced larger micelles [46]. In the case of **C14**, when DOX was encapsulated, the particle size increased significantly compared to the empty micelle, while in the case of **C16** and **C18**, no significant change was observed in DOX-encapsulated micelles and empty micelles. It was established that the size of indomethacin (IMC)-loaded micelles is larger than MePEG/ε-CL block copolymeric micelles without incorporating IMC [46]. We hypothesize that the significant increase in micelle size at **C14** is due to a weaker hydrophobic interaction between the drug and the polymer, which decreased the cohesive force in the inner core of the micelle and increased the micelle size. In addition, with respect to the size of the micelles, their volume at **C14** is the smallest, so the addition of DOX will also lead to an increase in the size of the micelles. For **C16** and **C18**, the addition of the drug did not significantly affect the hydrophilic–hydrophobic balance and no significant increase in micelle size was observed. The particle size distribution of DOX-encapsulating micelles was monodisperse (Figure 2) and the size was sufficient to exhibit the EPR effect.

The polydispersity of the empty micelles of **C16** and those incorporating DOX is the lowest.

#### 2.4.2. Drug Loading and Encapsulation Efficiency for Doxorubicin

It is known that the loading and release of hydrophobic drugs are determined by the hydrophobic blocks via the similar-to-similar interaction. Increasing the hydrophobicity of the polymer is known to increase the hydrophobic interaction with the drug and increase the rate of encapsulation. The end groups on the hydrophobic blocks inside the micellar core strongly dominated the drug loading and drug release [47]. The highest drug loading and encapsulation efficiency were obtained for **C16**-poly[hexadecylpoly(ethylene glycol) phosphate-*b*-hexadecylpoly(ethylene glycol)lactate phosphate]- 3.20% and 57.4%, respectively (Table 2). 

### 2.5. Release Rate of Loaded Doxorubicin from Micelles

Drug diffusion and the disassembly of micelles are the methods of drug release from polymeric micelles [48]. Many factors can affect the release of drugs, such as the rate of drug release related to the rate of degradation of the polymer, the length of the micellar nucleus fragments, the physical state of the nucleus, the size of the drug molecule, the position of the drugs in micelles, and the drug-loading rate [49]. In our case, we assume that doxorubicin releases from micelles via both methods, because it is known that phosphoester bonds P-O-C and C-O-C are hydrolytically unstable. At acidic conditions, the α–carbon atom of the alkyl group with respect to the phosphorus atom is attacked and aliphatic alcohol is removed. Hydrolysis of the alkyl group (side chain) proceeds faster, whereas in the strongly basic and neutral solutions, both the main chain and side groups proceed slower. This difference is related to the different mechanisms of hydrolysis prevailing at a given pH. At basic conditions, it is the phosphorus atom that is attacked by the strong nucleophile, and then the corresponding bond is broken, resulting in decreasing in the molecular weight of the polymer [50]. 

**Figure 3:**
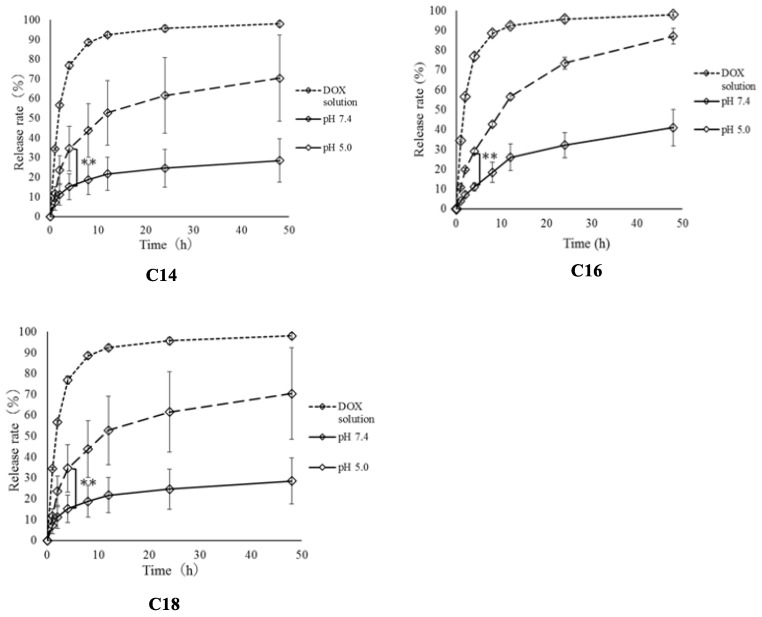
The cumulative release rate of DOX using C14, C16, and C18 polymers. (*n* = 3, mean ± S.D., ** *p* < 0.01,Tukey’s test).

It was established that DOX-containing micelles prepared from any polymer had a high sustained release of less than 35% in 48 h at pH 7.4 (Figure 3). Furthermore, at pH 5.0, DOX-containing micelles prepared from any polymer released more than 70% of DOX in 24 h and more than 90% in 48 h, confirming that the release was faster than at pH 7.4. This is because under acidic conditions, hydrolysis of the phosphoester bond in the side chain (hydrophobic block) is broken, causing micelles decomposition, resulting in the release of DOX. The result shows that the release rate of DOX increased from 35% to 80%, thus demonstrating that the poly[alkylpolyethylene glycol) phosphate-*b*-alkylpoly(ethylene glycol)lactate phosphate] DOX system has a pH-sensitive response capability. The results obtained showed that the release rate of doxorubicin is the same for all three polymers. The explanation is that the difference in molecular masses is relatively small, about 1000 units.

Release profiles of doxorubicin revealed that release proceeds in two stages: first stage—with a higher release rate from 0 h to 12 h; second stage—with a lower rate from 12 h to 40 h. This is observed at both pH 7. 4 and 5.0. It can be assumed that the decrease in the release rate of doxorubicin is due to their location in the micelle. A higher release rate implies that they are located in the core–corona interface or the periphery of the core, while a lower release rate means they are located in the center of the core. When drug molecules are predominantly located in the core, the higher the concentration of the drug, the slower the release rate [20]. 

### 2.6. Cytotoxicity Test 

**Figure 4:**
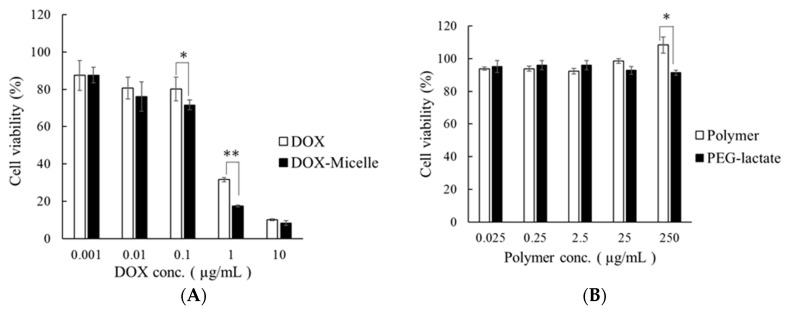
Cell viability of B-16 melanoma cells treated with DOX and DOX-micelle (**A**), and polymer and Peg-lactate (**B**) (*n* = 3, mean ± SD, * *p* < 0.05, ** *p* < 0.01, *t*-test).

DOX and DOX-micelle confirmed comparable cytotoxicity (Figure 4A).

As shown in Figure 4B, when the cell viability was calculated for each concentration, cytotoxicity was not observed at any concentration. Based on these results, it was determined that the toxicity of DOX-encapsulated micelles was due to the drug, not the base material. From the above, it was suggested that side effects could be reduced by micelle formation.

### 2.7. Cell Uptake Test Using Confocal Laser Microscopy (CLSM)

**Figure 5:**
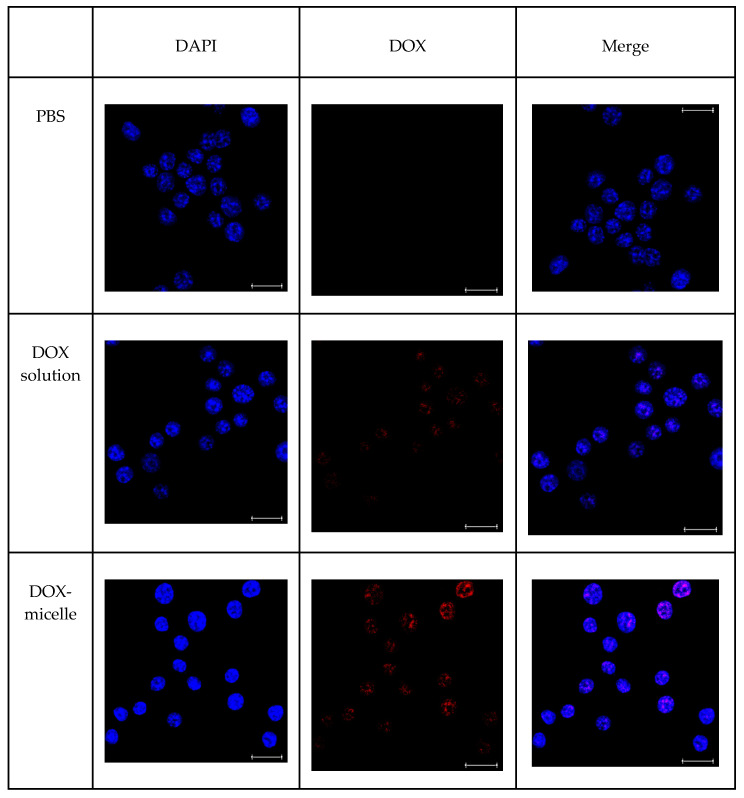
CLSM images of B-16 melanoma cells treated with DOX and DOX-micelle (scale bar, 5 µm).

Figure 5 shows an image of a cell uptake test using a CLSM. The result confirmed the cellular uptake of DOX and DOX-micelle. It was confirmed that the micelles prepared from the new polymer did not inhibit cellular uptake.

### 2.8. Cell Uptake Test Using Flow Cytometry (FACS)

**Figure 6:**
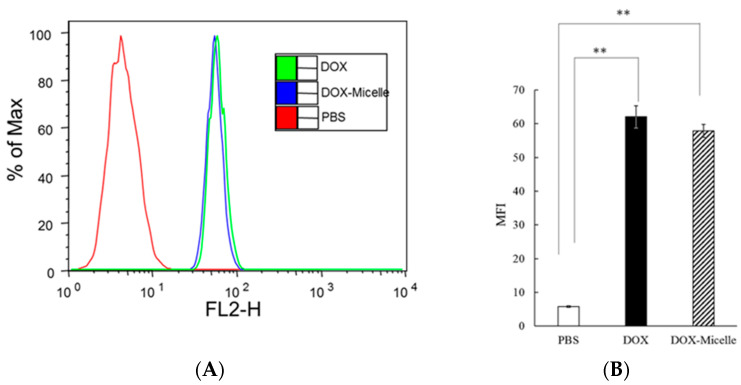
FACS histogram of B-16 melanoma cells treated with DOX and DOX-micelle (**A**), mean fluorescence intensity (MFI) (**B**) (*n* = 4, mean ± SD, ** *p* < 0.01, Tukey’s test).

The results of cell uptake evaluation using flow cytometry (FACS) are shown in Figure 6A. 

The results of cell uptake evaluation using flow cytometry (FACS) are shown in Figure 6A. This is a cell number frequency distribution (histogram) obtained by expressing the fluorescence intensity on the horizontal axis and the cell count number on the vertical axis as % of max. In addition, Figure 6B shows a graph of the mean values obtained from the measured values of fluorescence intensity for each group. The results of A and B, DOX and DOX-micelle are equivalent and they correlated with the results observed using the confocal laser microscope.

### 2.9. Pharmacokinetics Test Using Prepared Micelles

**Figure 7:**
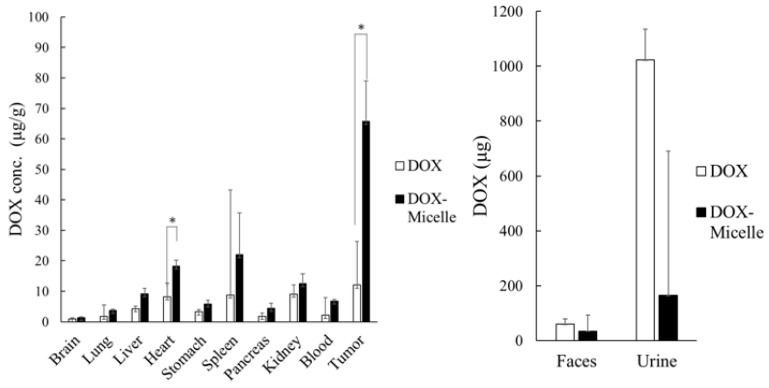
Biodistribution of DOX in tumor-bearing mice 24 h after treatment with free DOX and DOX-micelle (*n* = 3, * *p* < 0.05, *t*-test).

Inside the tumor, DOX-micelle had a significantly higher amount of DOX than a simple DOX solution (Figure 7). 

This is obviously due to the following: (i) DOX-micelle has a particle size of about 100 nm, which avoids renal excretion and makes it easier to enter the tumor; (ii) the present micelles are pH-sensitive and they selectively accumulated into the tumor, where pH is acidic. Additionally, a significantly higher amount of DOX was detected in the heart, which is thought to be due to the blood in the heart. From the above results, it was confirmed that the DOX-micelle prepared accumulates more DOX in tumors and less in normal tissues by avoiding renal excretion, compared to a simple DOX solution. It was suggested that the DOX-micelle prepared in this study may reduce the distribution to normal tissues and the occurrence of side effects, which are major problems with anticancer drugs.

### 2.10. Comparative Analysis

Based on our initial study [16], we select poly[hexadecylpoly(ethylene glycol) phosphate-*b*-hexadecylpoly(ethylene glycol)lactate phosphate], which with the highest drug loading and encapsulation efficiency at both ratios between number of blocks **A**:**B**, m:q ~1:1 and 8:1.

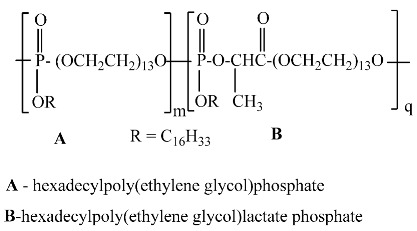



The molecular weight (Mn) of poly[hexadecylpoly(ethylene glycol) phosphate-*b*-hexadecylpoly(ethylene glycol)lactate phosphate] at a ratio of 1:1 is 16.8 × 10^3^ g/mol [16] and at a ratio of 8:1 is 16.1 × 10^3^ g/mol. The total molecular weight is the same; the difference is the molecular weights of the blocks. At a ratio of 1:1 molecular weight (Mn) of block A is 8.2 × 10^3^ g/mol and block B is 8. 6 × 10^3^ g/mol. At a ratio of 8:1 molecular weight (Mn) of block A is 14.2 × 10^3^ g/mol and block B is 1.9 × 10^3^ g/mol. At a ratio of 1:1 the highest drug loading and encapsulation efficiency—1.63% and 45.8%, respectively [16]. At a ratio of 8:1, the highest drug loading and encapsulation efficiency was—3.20% and 57.4%, respectively. The drug loading is two times higher, and the only difference is in molecular weights of blocks A and B. The results obtained revealed that the ratio between molecular weights of blocks of diblock polymer influences drug loading and encapsulation efficiency. 

## 3. Materials and Methods

### 3.1. Materials 

Poly(ethylene glycol)s with a number-average molecular weight of 600 g/mol was purchased from Wako Pure Chemical Industries, Ltd. (current FUJIFILM Wako Pure Chemical Co., Osaka, Japan). It was dried before use by a two-stage process: an azeotropic distillation with toluene and a subsequent 4 h heating at 120 °C under a dynamic vacuum. Ethyl lactate was purchased from Wako Pure Chemical Industries, Ltd. It was distilled before use. Sodium methoxide was purchased from Wako Pure Chemical Industries, Ltd. and was used as received. Diphenyl H-phosphonate was purchased from Wako Pure Chemical Industries, Ltd. with, a purity of 85%, containing <15% phenol. Phenol was removed by distillation before use. 1-Tetradecanol Mw = 214.30 g/mol, 1-hexadecanol Mw = 242.44 g/mol, 1-octadecanol Mw = 270.49 g/mol were purchased from Wako Pure Chemical Industries, Ltd. Trichloroisocianuric acid, 97% (TCIA) Sigma-Aldrich (St. Louis, MO, USA). 

### 3.2. Characterization Methods and Instruments 

All ^1^H, ^13^C, ^31^P{H} and ^31^P NMR spectra were recorded on a Bruker NMR spectrometer operating at 600 MHz at 37 °C in CDCl_3_. The molecular weights and polydispersity index (PDI) of polymers were determined by size exclusion chromatography (SEC) system (SIL-20A, RID10A, LC-20AD, CTO-20A, and DGU-20A3; Shimadzu Corp., Kyoto, Japan) with columns (GPC KF-804Lx2; Showa Denko K. K., Tokyo, Japan) using the PEG standard. THF, 10 mM at 40 °C was used as eluent (flow rate: 1.0 mL/min). 

MW-assisted transesterification reaction was carried out in an open vessel in a Milestone ROTO SYNTH rotative solid phase microwave reactor (Milestone, Sorisole, Italy). The device is equipped with a magnetic stirrer, an IR thermometer, and a magnetron with a frequency of 2.45 GHz with a maximum microwave power of 1200 W.

### 3.3. Microwave Synthesis of Poly(ethylene glycol)lactate

PEG600 (53.2 g, 8.8 × 10^−2^ mol) and ethyl lactate (5.2 g, 4.4 × 10^−2^ mol) were added to a two-necked round-bottomed flask, equipped with a magnetic stirring bar, inlet adaptor for inert gas, and vacuum condenser. The reaction was carried out under microwave irradiation conditions at 40 °C for 8 h under power (90 W) and reduced pressure (350 mmHg). Then, a reduced pressure treatment (0.8 mmHg) was carried out at 120 °C for 1 h, nitrogen was sealed in a two-necked flask, and the reaction was stopped. After that, the reaction mixture was subjected to a vacuum (1 mm Hg) at 60 °C. The yield of poly(ethylene glycol)lactate was 94% (27.8 g 4.1 × 10^−2^ mol).

^1^HNMR (CDCl3), δ (ppm) (Figure S1): 1.43 (d, C*H*_3_CH, 3 J(H,H) = 7.07 Hz); 1.428 (d, C*H*_3_CH, ^3^J(H,H) = 6.83 Hz); 3.47 (d, CH_3_CH(O*H*), ^3^J(H,H) = 4.6 Hz); 3.64, s, -C*H*_2_OCH_2_-; 3.72 (t, *H*OCH_2_-, ^3^J(H,H) = 4.6 Hz); 4.28 to 4.34 area for CH_3_C*H*(OH)C-, quartet, and CH_2_C*H*_2_OC(O)-, triplet, overlapping of the signals; ^13^C{H}NMR (CDCl_3_), δ (ppm) (Figure S2): 20.34, *C*H_3_CH; 61.64, HO*C*H_2_-; 64.40, CH_3_*C*H-; 66.72, -C(O)OC*H*_2_-; 68.83, -C(O)O*C*H_2_CH_2_O-; 70.28, HO*C*H_2_CH_2_OCH_2_-; 70.51, -(O*C*H_2_CH_2_O)-; 72.48, HOCH_2_CH_2_O-; 175.51, C=O. At a poly(ethylene glycol)lactate yield of 94.0%, the reaction mixture contains poly(ethylene glycol)lactate 27.8 g, 0.041 mol, 49.3%; PEG 600 28.6 g, 0.048 mol, 50.7%. The molar ratio between poly(ethylene glycol)lactate and PEG 600 is 1:1.2. 

### 3.4. Synthesis of Poly[poly(ethylene glycol) H-phosphonate]-b-[poly(ethylene glycol)lactate H-phosphonate]

A 10.8 g reaction product from Section 3.3. containing 49.3% poly(ethylene glycol 600)lactate 5.32 g, 0.008 mol) and 50.7% PEG600 (5.48 g, 0.009 mol) was added to 4.68 g, 0.016 mol 85% diphenyl H-phosphonate under Ar-atmosphere in a round-bottomed two-necked flask, equipped with a magnetic stirrer, condenser attached to the vacuum line. The reaction was carried out at a temperature of 135 °C for 6 h, 160 °C for 4 h, and 185 °C for 3 h, vacuum 0.6 mm Hg. The progress of the reaction was monitored by the amount of phenol that evolved. When the evolution of phenol stopped, the system was cooled under argon flow. The reaction product was dissolved in methanol with stirring at room temperature. Diethyl ether was added. Two phases were formed. The lower phase was dry. The product was obtained as a waxy solid. Yield 8.8 g, 56.16%.

^1^H NMR (CDCl_3_) (Figure S3) δ, ppm, reaction product after 10 h heating: δ = 1.42 ppm, d, ^3^J(H,H) = 7.07 Hz for -C(O)CH(C*H*_3_) protons; 1.60 ppm, d, ^3^J(H,H) = 6.83 Hz, POCH(C*H*_3_)C(O)-; 1.58 ppm, ^3^J(H,H) = 6.83 Hz, -POCH(C*H*_3_)C(O)-; 3.64, s, -C*H*_2_OC*H*_2_; 4.13–4.37 ppm, m, OCH_2_C*H*_2_OP(O)(H)OC*H*_2_CH_2_-. 4.99–5.06 ppm, m, POC*H*(CH_3_)-protons; 6.83–7.21 for aromatic protons; δ = 6.95 ppm, d, ^1^J(P,H) = 717.69 Hz. OCH_2_OP(O)(*H*)OCH_2_; δ = 7.02 ppm, d, ^1^J(P,H) = 729.40 Hz for OCH_2_OP(O)(*H*)OCH(CH_3_); δ = 7.13 ppm, d, ^1^J(P,H) = 734.8 Hz for OCH_2_OP(O)(*H*)OCH(CH_3_); δ = 7.16 ppm, d, ^1^J(P,H) = 724.76 Hz for PhOP(O)(*H*)OCH_2_-; ^31^P{H} NMR (Figure 4) (CDCl_3_), δ ppm,: 9.31 ppm with integral intensity (II) = 1.00; 8.62 ppm, II = 0.06; 7.32 ppm, II = 0.06; 6.85 ppm, II = 0.06 and 5.37 ppm, II = 0.12. The ratio between the integral intensity of the signals at 9.31 ppm and (8.62 ppm and 7.32 ppm) is 1: 0.12 = 8.33. ^31^P NMR (CDCl_3_) (Figure S4), δ ppm: 9.31 ppm, doublet of quintets with ^1^J(P,H) = 737.6 Hz, and ^3^J(P,H) = 10.0 Hz, CH_2_O*P*(O)(H)OCH_2_; 8.62 ppm, dq, with ^1^J(P,H) = 751.98 Hz, and ^3^J(P,H) = 7.47 Hz and 7.23 dq, with ^1^J(P,H) = 747.0 Hz, and ^3^J(P,H) = 7.47 Hz, CH_2_O*P*(O)(H)OCH(CH_3_)-. 6.85 ppm, dt, with ^1^J(P,H) = 727.0 Hz, HO-*P*(O)(H)OCH_2_; 5.37 ppm, dt, with ^1^J(P,H) = 744.51 Hz, and ^3^J(P,H) = 10.0 Hz, PhO-*P*(O)(H)OCH_2_-.

### 3.5. Synthesis of Poly[alkylpoly(ethylene glycol) phosphate-b-alkylpoly(ethylene glycol)lactate phosphate]s

#### 3.5.1. Synthesis of Poly[hexadecylpoly(ethylene glycol) phosphate-*b*-[hexadecylpoly(ethylene glycol)lactate phosphate]

Poly[hexadecylpoly(ethylene glycol) phosphate)-*b*-hexadecylpoly(ethylene glycol)lactate phosphate] was obtained from poly[poly(ethylene glycol) H-phosphonate-*b*-poly(ethylene glycol)lactate H-phosphonate], trichloroisocyanuric acid and 1-hexadecanol in a one-pot synthesis. The entire synthesis was carried out under an inert atmosphere. In a round-bottomed tri-necked flask, equipped with a magnetic stirrer, a reflux condenser was put 1.59 g, 2.4 × 10^−3^ mol poly[poly(ethylene glycol) H-phosphonate)-*b*-poly(ethylene glycol)lactate H-phosphonate] synthesized in item from Section 3.4. and dissolved by adding 10 mL of acetonitrile. Trichloroisocyanuric acid (Mw 232.41 g/mol) (0.57 g, 2.4 × 10^−3^ mol) dissolved in 5 mL of acetonitrile was added. The reaction mixture was kept for 5 h at 40 °C, 4 h at 60 °C, and 5 h at 50 °C. The optimum reaction time of 14 h was determined by observing ^31^P{H} NMR spectroscopy. ^31^P{H}NMR spectrum showed signals at 5.46; 4.95; 4.36; 0.24 and −0.08 ppm (Figure S5) which are characteristic of chlorophosphate structures. The ratio between the integral intensity of the signals at 5.46 ppm and those of the signals at 4.95 ppm and 4.36 ppm is 1:0.09. To the reaction product, poly[poly(ethylene glycol) chlorophosphate-*b*-poly(ethylene glycol)lactate chlorophosphate] without isolation, 1-hexadecanol (Mw = 242.44 g/mol) 0.58 g (2.4 × 10^−3^ mol in 7 mL diethyl ether was added and the mixture was stirred at 50 °C for 24 h under nitrogen conditions. The obtained polymer was dissolved in methanol with stirring at room temperature, and allowed to stand for one day at 5 °C to precipitate cyanuric acid, as a byproduct. Thereafter, the solution was filtered using a filter paper with a pore size of 5 µm and a syringe filter with a pore size of 0.45 µm in the cold room, and the solvent was completely removed using an evaporator. Then, the desired polymer was obtained by drying under reduced pressure overnight. Yield 2.17 g, 97.0%.

^31^P{H}NMR (CDCl_3_), δ (ppm) −0.75; −1.63; −5.94 and −12.54.

#### 3.5.2. Synthesis of Poly[tetradecylpoly(ethylene glycol) phosphate-*b*-tetradecylpoly(ethylene glycol)lactate phosphate] 

Using the same procedure, the following was synthesized: poly[tetradecylpoly(ethylene glycol) phosphate-*b*-tetradecylpoly(ethylene glycol)lactate phosphate]. A typical synthetic procedure yielded poly[poly(ethylene glycol) H-phosphonate-*b*-poly(ethylene glycol)lactate H-phosphonate] 1.72 g, 2.65 × 10^−3^ mol; trichloroisocyanuric acid (Mw 232.41 g/mol) 0.62 g, 2.65 × 10^−3^ mol; 1-tetradecanol (Mw = 214.39 g/mol) 0.57 g, 2.65 × 10^−3^ mol. Yield 2.14 g, 94.0%.

^31^P{H}NMR (CDCl_3_), δ (ppm); −0.73; −1.60; −5.6 and −11.04.

#### 3.5.3. Synthesis of Poly[octadecylpoly(ethylene glycol) phosphate)-*b*-octadecylpoly(ethylene glycol)lactate phosphate]

Using the same procedure, the following was synthesized: poly[octadecyl poly(ethylene glycol) phosphate-*b*-octadecylpoly(ethylene glycol)lactate phosphate]. A typical synthetic procedure yielded poly[poly(ethylene glycol) H-phosphonate-*b*-poly(ethylene glycol)lactate H-phosphonate] 2.18 g, 3.32 × 10^−3^ mol; trichloroisocyanuric acid (Mw 232.41 g/mol) 0.77 g, 3.32 × 10^−3^ mol; 1-octadecanol (Mw = 270.49) 0.9 g, 3.32 × 10^−3^ mol. Yield 2.93 g, 96.0%. 

^31^P{H}NMR (CDCl_3_), δ (ppm) −0.74 ppm; −1.58 ppm, −5.63 ppm, −5.2 ppm, and −11.15 ppm. 

### 3.6. Measurement of Polymeric Micelle Size 

An amount of 50 mg of the synthesized poly[alkylpoly(ethylene glycol) phosphate-*b*-alkylpoly (ethylene glycol)lactate phosphate]s (referred to as sample) and 20 mL of acetone were added to the flask and dissolved completely. The tin film was formed. Then, the remaining acetone was completely removed by drying under reduced pressure. A PBS solution of pH 7.4 was added thereto. Then, after ultrasonic irradiation, it was filtered through a 0.2 µm filter. Furthermore, the particle size was measured at 37 °C by dynamic light scattering (DLS) system, ELS-Z2 (Otsuka Electronics Co., Ltd., Hirakata, Japan), to evaluate dispersibility.

### 3.7. Preparation of Doxorubicin-Loaded Micelles

Two mg of doxorubicin hydrochloride was weighed into a flask, 0.2 mL of triethylamine was added, and dehydrogenation was performed. Amounts of 50 mg of sample and 20 mL of chloroform were added thereto and completely dissolved. Thereafter, evaporation was performed to form a thin film, and chloroform was completely removed by drying under reduced pressure. A PBS solution was added there. After ultrasonic irradiation, it was filtered with a 0.2 μm filter to remove released DOX. Furthermore, to confirm micelle formation, particle size was measured using DLS. Hereinafter, the DOX-containing micelle will be referred to as DOX-micelle.

### 3.8. Measuring the Drug Loading and Encapsulation Efficiency of Micelles for Doxorubicin

Micelles containing 2.0 mg of DOX were prepared in the same manner as in Section 3.7, and 2 mL of the micelles were freeze-dried. Thereafter, it was dissolved in a mobile phase (pH 2.5 PBS solution: Acetonitrile = 2:1) and filtered using a 0.20 μm filter to remove precipitated salts. Then, the peak of the micelle solution was detected using high-performance liquid chromatography (HPLC), and DOX was quantified using a separately prepared calibration curve. The measurement conditions were a measurement wavelength of 254 nm, a column temperature of 30 °C, a flow rate of 0.5 mL/min, an injection volume of 20 μL, and an analysis time of 10 min. The mobile phase was acetonitrile: phosphate buffer 0.01 M (pH 2.5) = 1:2 was used. The drug loading and encapsulation efficiency were calculated using the following formulas.

$$\mathrm{Drug}\;\mathrm{loading}(\%)=\frac{Amount\;of\;DOX\;in\;micelles\;(mg)}{Amount\;of\;micelles\;after\;freeze-drying(mg)}\times 100$$

$$Encapsulation\;efficiency\left(\%\right)=\frac{Amount\;of\;DOX\;in\;micelles\left(mg\right)}{Amount\;of\;DOX\;added\;during\;preparation\left(mg\right)}\times 100$$

### 3.9. Evaluation of Release Properties of Doxorubicin-Encapsulated Micelles

We put 5 mL of the DOX-containing micelle solution prepared in item 5.2.3.1 into a dialysis membrane, and added 95 mL of pH 7.4 PBS (−) solution or pH 5.0 acetate buffer to a vial at 37 °C for a predetermined time (1, 2, 4, 8, 12, 24, 48 h) with water-bath shaking [51]. At this time, the external solution was completely replaced. Thereafter, the external solution was measured by HPLC and the release rate was calculated.

### 3.10. Cytotoxicity Test Using WST-8 Assay

B16 melanoma cells were cultured in DMEM supplemented with 10% inactivated FBS (37 °C, 5% CO_2_). B16 melanoma cells were seeded in 100 μL each at 5.0 × 10^3^ cells in a 96-well plate and incubated for 24 h. Then, 10 μL of Doxorubicin and DOX-micelle (DOX concentration 0.01, 0.1, 1, and 10 μg/mL) were added and incubated for 24 h. Thereafter, 10 μL of CCK-8 solution was added, and after a color reaction was performed for 1 h, the absorbance was measured using a microplate reader, and the cell survival rate was calculated. To the micelle, we also added 10 µL polymer of poly(ethylene glycol lactate) at concentrations of 0.25, 2.5, 25, and 250 µg/mL, incubated for 24 h, and cell viability was calculated in the same manner. The cell experiments were conducted with reference to the protocol of Dojindo Kagaku Kenkyusho Co., Ltd. (Tokyo, Japan).

$$\mathrm{Cell}\;\mathrm{viability}(\%)=\frac{ABS\left(sample\right)-ABS(control)}{ABS\left(PBS\right)-ABS(control)}\times 100$$

### 3.11. Cell Uptake Test Using Confocal Laser Microscopy (CLSM)

Cell uptake was observed using DOX-micelle. B16 melanoma cells, which are mouse-derived malignant melanoma cells, were seeded on a culture slide with a chamber at a concentration of 1.0 × 10^5^ cells/mL, and then incubated for 24 h under conditions of 5% CO_2_/air and 37 °C, and the cells were plated. Doxorubicin and DOX-micelle (5.0 µg/mL) were each seeded and incubated for 2 h. Thereafter, the supernatant was discarded, and particles adhering to the cell surface were washed with PBS (−), and 4% paraformaldehyde was added for fixation. After adding TritonX100 (0.1%) and washing, staining was performed with 50 µL/well DAPI. This was washed, dried, and observed using a confocal laser microscope. The fluorescence intensity of Doxorubicin was set between 488 and 575 nm, and the fluorescence intensity of DAPI was set between 345 and 455 nm [52].

### 3.12. Cell Uptake Test Using Flow Cytometry (FACS)

B16 melanoma cells were seeded in a 6-well plate at a concentration of 1.0 × 10^5^ cells/mL, and then incubated for 24 h under conditions of 5% CO_2_/air and 37 °C to allow the cells to adhere to the plate. Doxorubicin and DOX-micelle (5 µg/mL) were added and incubated for 2 h at 37 °C and 5% CO_2_/air. Thereafter, particles adhering to the cell surface were washed with PBS (−), subjected to a deadhesion treatment using EDTA, and then centrifuged at 300 g for 10 min. After removing the supernatant, the cells were dispersed in FACS buffer (0.3% BSA aq.), and the fluorescence intensity of Doxorubicin was measured using FACSCalibur. The fluorescence intensity of Doxorubicin was measured under the same conditions as in Section 3.11.

### 3.13. Pharmacokinetics Study Using Prepared Micelles

B16 melanoma cells (1.0 × 10^6^ cells/head) were subcutaneously transplanted into the right hind limb of a ddY mouse (4 weeks old, male) to create a tumor-bearing mouse model. In addition, B-16 melanoma cells were dispersed in PBS, and the subcutaneous administration volume was 50 μL. For the pharmacokinetics study, mice with an estimated tumor burden of approximately 80 mg were calculated using the formula, described by Rapoport et al. [53]. Doxorubicin and DOX-micelle (10 mg/kg) were administered to the prepared tumor-bearing mice (7 weeks old) through the tail vein, kept in a metabolic cage (Techniplast Japan Co., Ltd., Tokyo, Japan) for 24 h, and then sacrificed. Then, each organ, blood, tumor, urine, and feces were collected, and after protein removal, the amount of DOX was measured using HPLC. The protein removal operation was performed as follows. An amount of 0.8 mL of physiological saline was added to each organ, and the organs were completely crushed using a homogenizer. Next, 2.5 mL of acetonitrile was added, vortexed for 30 s, centrifuged (4000 rpm, 20 min), and 900 µL of supernatant was collected. It was then concentrated by removing the solvent with a nitrogen purge, dissolved in 450 µL of developing solvent, and then measured using HPLC [54,55,56]. In addition, measurements were made using methyl *p*-benzoate as an internal standard substance, and the concentration was calculated by the internal standard method. The animal experiments in this study were conducted with the approval of the Tokyo University of Science Experimental Animal Ethics Committee.

## 4. Conclusions 

pH-sensitive amphiphilic diblock polyphosphoesters containing lactic acid units were synthesized by multistep one-pot polycondensation reactions. The inclusion of lactic acid leads to an increase in the hydrophilicity of the copolymer due to the carbonyl group. The newly synthesized polymers self-assembled into the micellar structure in an aqueous solution. The effects of the molecular weight of the copolymer and the length of the hydrophobic chain on micelle formation and stability and micelle size were studied via dynamic light scattering (DLS). Drug loading and encapsulation efficiency tests using doxorubicin revealed that hydrophobic drugs can be delivered by copolymers. The highest drug loading and encapsulation efficiency were obtained when a hydrophobic alcohol was used hexadecanol—3.2% and 57.4%, respectively. The poly[alkylpoly(ethylene glycol) phosphate-*b*-alkylpoly(ethylene glycol)lactate phosphate] DOX system demonstrate pH-sensitive capabilities, resulting in selective release of DOX in acidic tumor environments. The results obtained indicate that amphiphilic diblock polyphosphoesters have potential as drug carriers.

## Figures and Tables

**Table 1:** Particle size and polydispersity index of micelles (*n* = 3, mean ± S.D.).

Polymer	Micelle Size (nm)	DOX-Micelle Size (nm)	Micelle PDI	DOX-Micelle PDI
C14	71.0 ± 48.9	116.9 ± 53.8	0.257 ± 0.02	0.230 ± 0.02
C16	105.3 ± 45.3	119.9 ± 47.6	0.182 ± 0.01	0.200 ± 0.01
C18	86.4 ± 43.4	100.2 ± 41.1	0.231 ± 0.02	0.240 ± 0.002

**Table 2:** Characterization of DOX-micelles (*n* = 3, mean ± S.D.).

Polymer	Yield (%)	Drug Loading (%)	Encapsulation Efficiency (%)
C14	78.1 ± 5.9	1.5 ± 0.2	31.4 ± 2.0
C16	89.8 ± 8.3	3.2 ± 0.3	57.4 ± 3.2
C18	84.2 ± 7.0	2.4 ± 0.2	52.9 ± 0.1

## Data Availability

The data presented in this study are available in the Supplementary Materials.

## Supplementary Materials

The supporting information can be downloaded at: https://www.mdpi.com/article/10.3390/ijms25084518/s1.
