# Supplementary material for: pH-Sensitive Amphiphilic Diblock Polyphosphoesters with Lactate Units: Synthesis and Application as Drug Carriers
Source: Int J Mol Sci. 2024 Apr 20;25(8):4518. doi: 10.3390/ijms25084518 (PMC11049995; doi:10.3390/ijms25084518)
Supplement: Supplementary file 1 [file ijms-25-04518-s001.zip › ijms-2938311-supplementary.pdf]

pH - Sensitive Amphiphilic Diblock Polyphosphoesters with Lactate Units: Synthesis and Application as Drug Carriers

Kasumi Mochizuki[1], Violeta Mitova[3], Kimiko Makino[1], Hiroshi Terada[1†], Issei Takeuchi[1,2,] and Kolio Troev[1,3*]

[1] Faculty of Pharmaceutical Sciences, Tokyo University of Science, 2641, Yamazaki, Noda 278-8510, Chiba, Japan
[2] Faculty of Pharmaceutical Science, Josai International University, 1 Gumyo, Togane 283-8555, Chiba, Japan
[3] Institute of Polymers, Bulgarian Academy of Sciences, 1113 Sofia, Bulgaria
*Correspondence: ktroev@polymer.bas.bg (K.T.)
[†] Deceased author.

Supplementary data

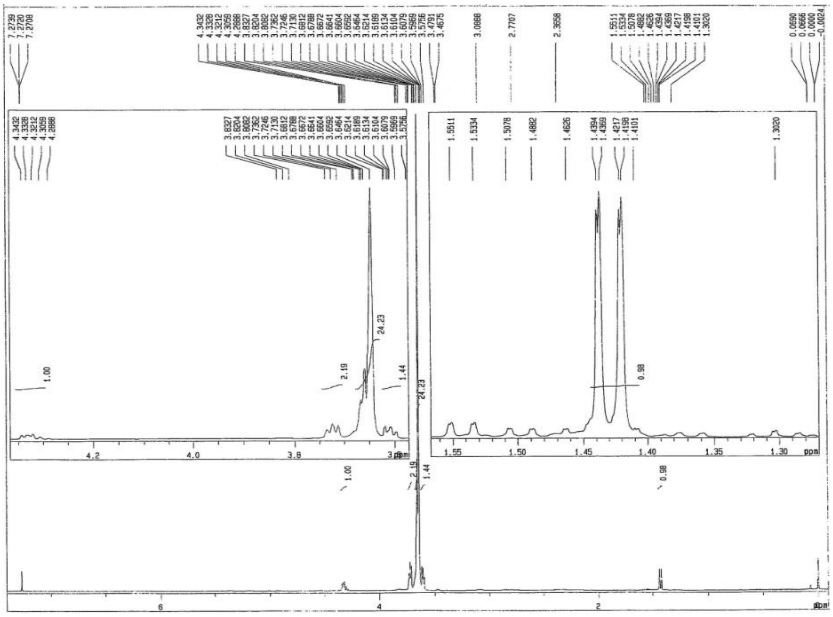

Figure S1. ^{1}H NMR spectrum of the reaction product obtained from poly(ethylene glycol) and ethyl lactate.

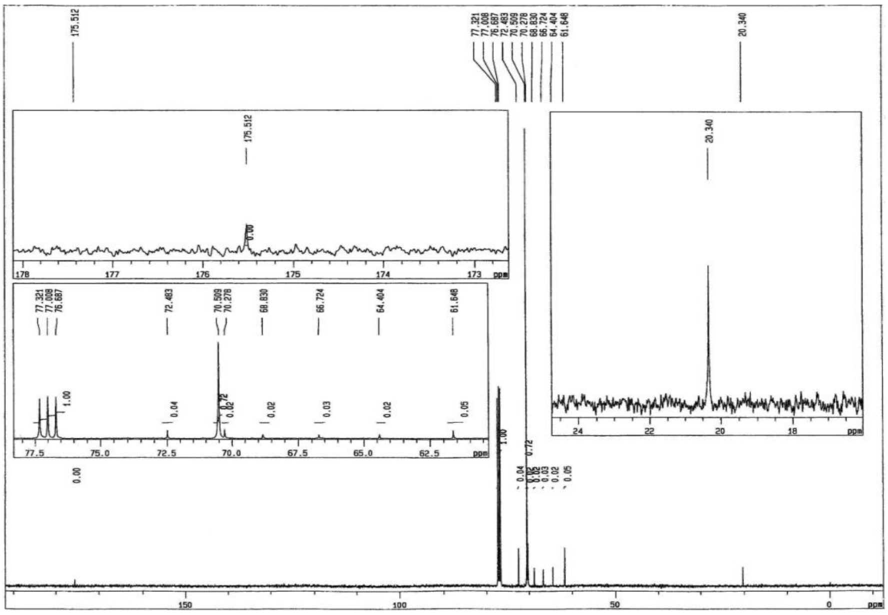

Figure S2. $^{13}C\{H\}$ NMR spectrum of the reaction product obtained from poly(ethylene glycol) and ethyl lactate.

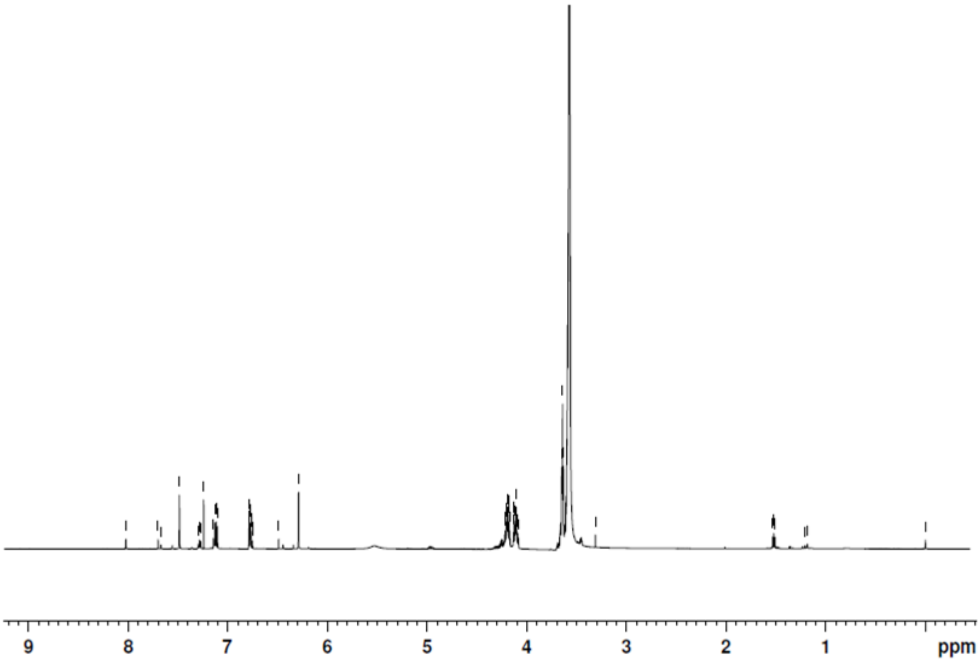

Figure S3. ^{1}H NMR spectra of poly[poly(ethylene glycol) H-phosphonate -*b*- poly(ethylene glycol)lactate H-phosphonate) lactate in $CDCl_3$.

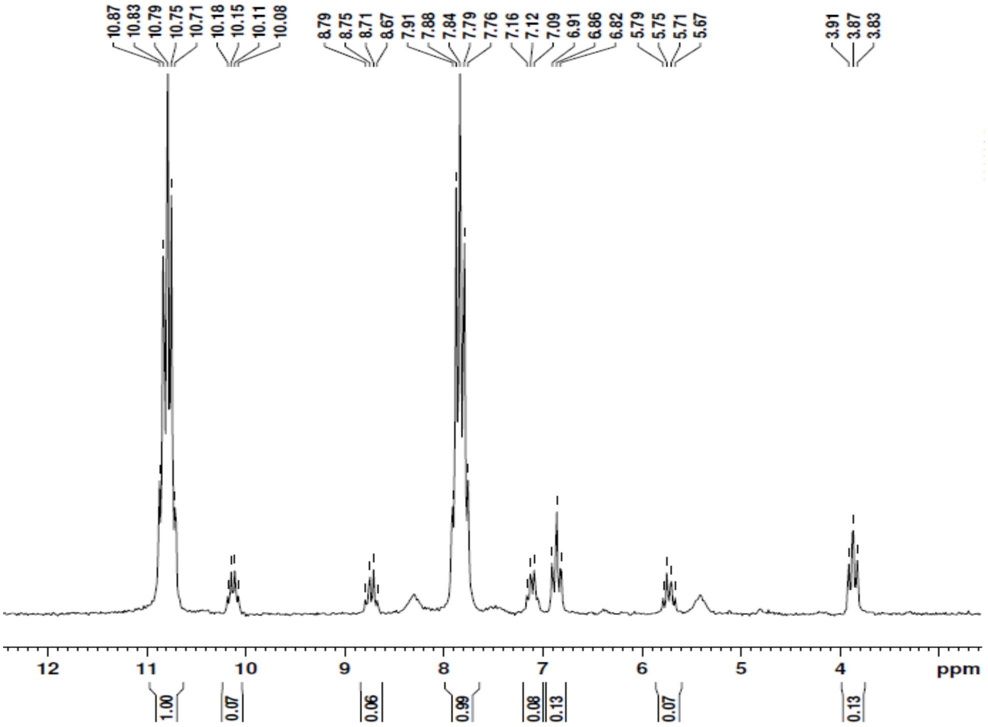

Figure S4. ^{31}P-NMR spectra of poly[poly(ethylene glycol) H-phosohonate-*b*-poly(ethylene glycol)lactate H-phosphonate] in $CDCl_3$.

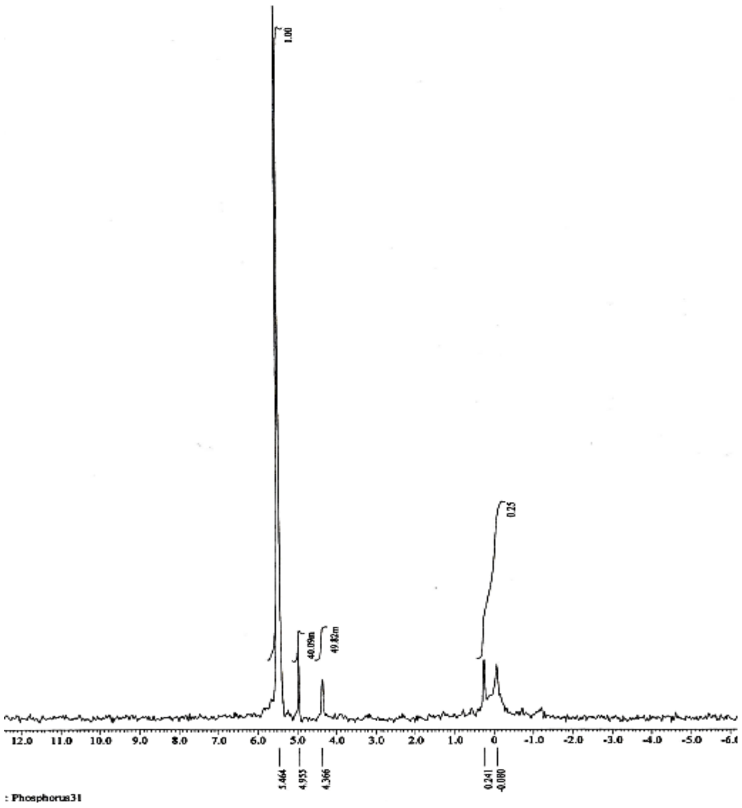

Figure **S5**. $^{31}P\{H\}$-NMR spectra of poly[poly(ethylene glycol Cl-phosphate-*b*-poly(ethylene glycol) lactate Cl-phosphate]in $CDCl_3$.

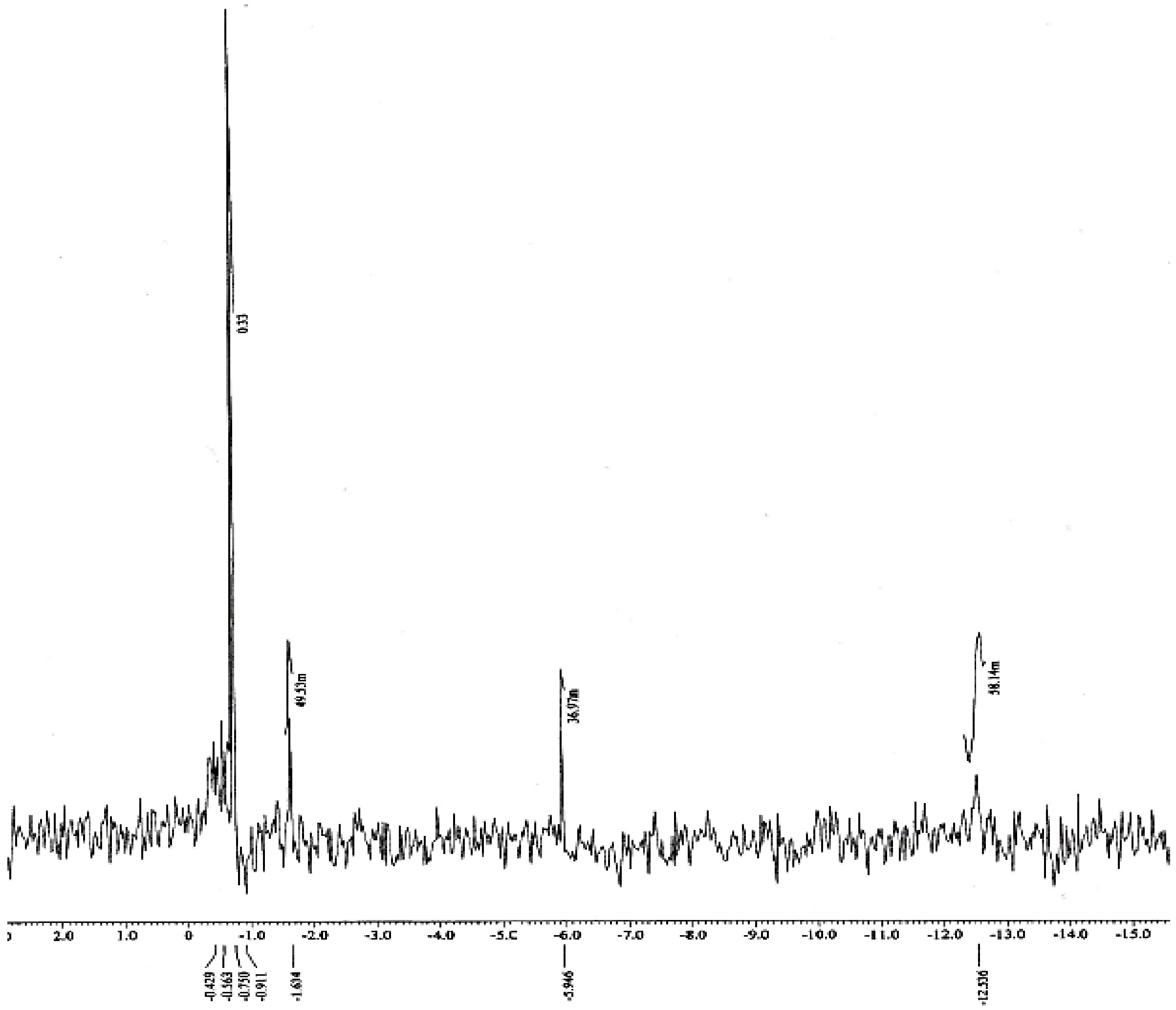

Figure S6. ^{31}P-NMR spectra of poly[hexadecylpoly(ethylene glycol) phosphate-b-hexadecylpoly(ethylene glycol)lactate phosphate] in $CDCl_3$

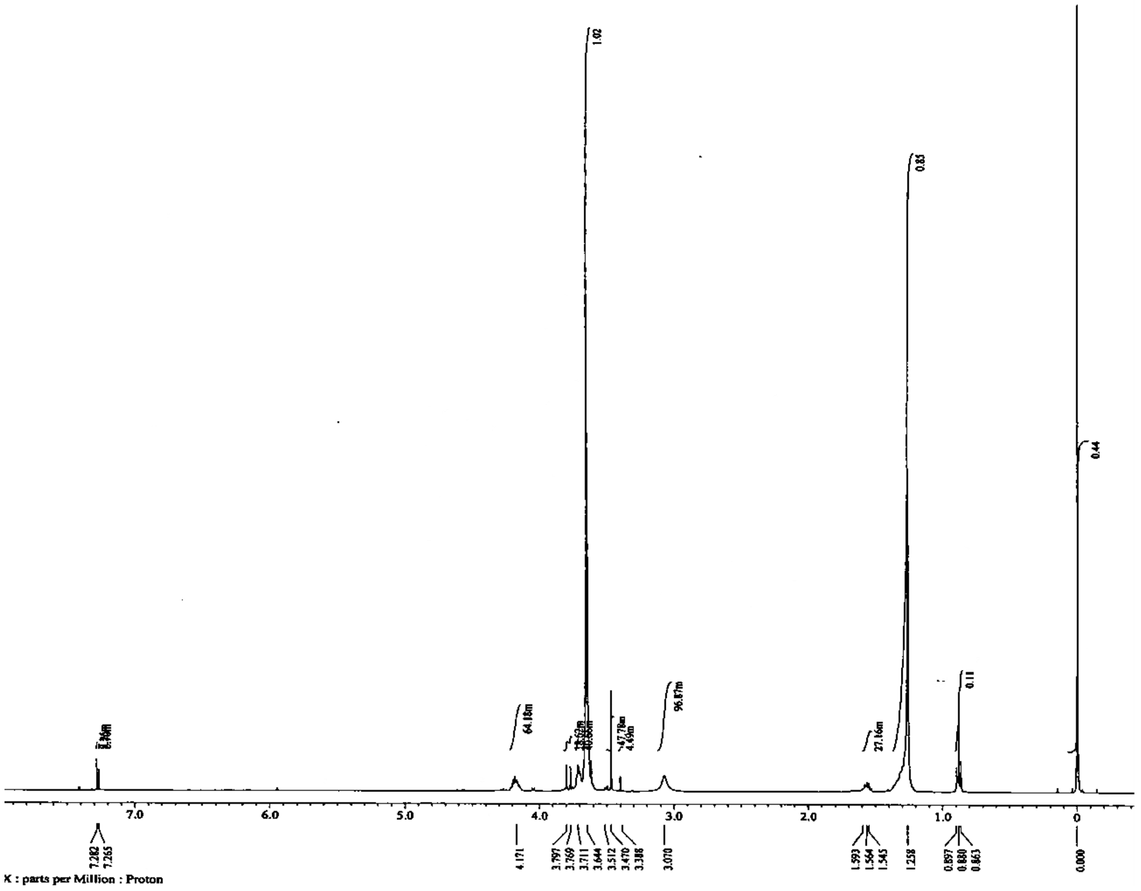

Figure S7. ^{1}H-NMR spectra of poly[hexadecylpoly(ethylene glycol) phosphate- *b*-hexadecylpoly(ethylene glycol)lactate phosphate) in $CDCl_3$.
